# The correlation between volumetric bone mineral density and morphological parameters of the proximal femur and clinical outcomes in ankylosing spondylitis patients with hip involvement

**DOI:** 10.1186/s12891-021-04912-3

**Published:** 2022-01-03

**Authors:** Xinfeng Wu, Liang Zhang, Tao Bian, Siliang Man, Hongchao Li, Wei Liu, Yixin Zhou

**Affiliations:** 1 Department of Spine Surgery, Beijing Jishuitan Hospital, Fourth Clinical College of Peking University, No. 31 Xinjiekou East Street, Xicheng District, Beijing, 100035 China; 2 Department of Orthopedic Surgery, Beijing Jishuitan Hospital, Fourth Clinical College of Peking University, No. 31 Xinjiekou East Street, Xicheng District, Beijing, 100035 China; 3 Department of Rheumatology and Immunology, Beijing Jishuitan Hospital, Fourth Clinical College of Peking University, No. 31 Xinjiekou East Street, Xicheng District, Beijing, 100035 China

**Keywords:** ankylosing spondylitis; bone mineral density; proximal femur; quantitative computed tomography

## Abstract

**Background:**

To measure volumetric bone mineral density (vBMD) with quantitative computed tomography (QCT) in the proximal femur of ankylosing spondylitis (AS) patients with hip involvement and analyze their correlations with radiographic and clinical parameters.

**Methods:**

Sixty-five AS inpatients were enrolled in this study. The bone mineral density was measured by QCT and dual-energy x-ray absorptiometry (DXA), respectively. The morphological parameters of the proximal femur were measured on digital anteroposterior (AP) radiographs of the pelvis. The correlations between them were analyzed by SPSS software.

**Results:**

The average trabecular vBMD measured at the femoral neck was 136.38 ± 25.58 mg/cm^3^. According to the BASRI-Hip score, group A consisted of 39 hips (0–2 score) and group B consisted of 26 hips (3–4 score). There were significant differences regarding trabecular CTXA equivalent T-score between group A and B at the femoral neck (*p* = 0.004); intertrochanteric region (*p* < 0.001) and greater trochanter (*p* = 0.001). The trabecular CTXA equivalent T-score at femoral neck had a negative correlation with disease duration (*r* = − 0.311, *p* = 0.012) and with CBR (*r* = − 0.319, *p* = 0.010).

**Conclusions:**

The low trabecular bone density at the site of the hip was associated with the duration of disease progression and degree of hip involvement. Meanwhile, it had a correlation with hip function status although we failed to confirm a significant relationship between hip vBMD and disease activity.

## Background

Hip involvement is common in ankylosing spondylitis (AS) and accounts for 50% of all affected joints [1–3]. Hip involvement may lead to an impaired physical function, psychosocial status and quality of life. Total hip arthroplasty (THA) is a reliable treatment of choice for patients with end-stage hip involvement. Unfortunately, hip prostheses usually have a limited lifespan, and revision surgery seems inevitable. Theoretically, a comprehensive system including early detection and intervention for hip involvement in this special patient population is anticipated. However, current definitions for hip involvement in AS make it difficult to differentiate hip involvement by inflammation and syndesmophyte from degenerative hip changes [4]. Moreover, studies of therapy in AS have been mainly focused on spine radiology and to some extent on peripheral arthritis or enthesitis. Little is known regarding the exact effects of traditional nonsteroidal anti-inflammatory drugs (NSAIDs), disease modifying antirheumatic drugs (DMARDs) or biological agents on the involved hip and the need for hip replacement surgery [4].

Osteoporosis (OP), in terms of decreased bone mineral density (BMD), is a meaningful feature of AS [5–7]. It is now well accepted that patients with AS have a higher prevalence of both osteopenia and OP. However, the reported prevalence of low BMD varies widely, ranging from 4 to 58% [8–17]. The explanations for this discrepancy include the paradoxical coupling of syndesmophyte formation combined with bone loss locating on spine and peripheral joints [18, 19] and a wide variation of techniques used to evaluate BMD. For AS patients with end-stage hip involvement, the decreased BMD at the site of hip was associated with a higher perioperative complication rate of THA as a salvage option, including blood loss, periprosthetic fracture and aseptic loosening of the implant [20–22]. However, there are few reports on a clear correlation between the changes of hip BMD and the anatomy of the proximal femur and clinical outcomes for AS patients with hip involvement on the varying stages.

The optimal method to identify BMD loss in AS patients remains controversial. Dual-energy x-ray absorptiometry (DXA) is the routine method of assessing BMD. However, the value obtained from DXA is the areal BMD including both cortical bone and trabecular bone. Consequently, some authors suggest that the best site to assess bone loss in AS patients is femoral neck or the lateral lumbar spine in order to avoid the interference from osteoproliferation of the lumbar spine [13, 15, 23, 24]. Quantitative computed tomography (QCT) has the advantage of measuring volumetric BMD (vBMD) without being affected by cortical artifacts and consequently is highly attractive to patients with AS, especially in stages of advanced ankylosis with substantial syndesmophyte [18, 25, 26]. The aims of this study were to: (1) compare the detection rate of the OP and osteopenia of proximal femur in AS patients by DXA and QCT and (2) assess the correlation between vBMD and anatomic parameters of the proximal femur and clinical outcomes of AS patients on different stages of hip involvement.

## Methods

### Patients

This was an observational, cross-sectional, single-center study. The outpatients who visited rheumatology and adult joint reconstruction surgery clinics from April 2017 to September 2019 were recruited. The study was approved by the local ethics committee (202004–08), and informed consent was obtained from each patient prior to participation in the study.

The inclusion criteria included: (1) diagnosis of AS according to the 1984 modified New York criteria; (2) patients with the age ranging from 18 to 55; (3) unilateral or bilateral hip pain and (or) limited range of motion (ROM). For patients with bilateral hip involvement, we selected the more severely involved side as the study subject. The exclusion criteria included: (1) history of congenital or childhood disease, surgery, deep infection, trauma, and tumor of the hip; (2) metabolic and endocrine diseases; (3) inflammatory arthritis and connective tissue disease other than AS; (4) the use of bisphosphonate, corticosteroids and biological agents; (5) female patients with menopause, and (6) pregnancy. With these criteria, a total of 65 outpatients with 65 affected hips were included.

### Patient demographics and clinical parameter

The patients completed questionnaires regarding demographics including sex, and body mass index (BMI); AS-related clinical information including age at outpatient visit, age at onset of AS, duration of AS, diagnosis delay, disease activity, functional status, extra-articular manifestations (EAMs) (current or past) including uveitis, psoriasis, and inflammatory bowel disease (IBD), family history, smoking habits (current or past), and medication status. The use of medications including NSAIDs and DMARDs was recorded, with patients who had taken treatment agents for periods of 1 year or longer being considered as sustained users. Disease activity and functional status were assessed using the Bath ankylosing spondylitis disease activity index (BASDAI) [27] and the Bath ankylosing spondylitis functional index (BASFI) [28], respectively. The patient-reported outcomes (PROs), were assessed by using the Short Form-12 (SF-12) and Ankylosing Spondylitis Quality of Life (ASQoL) scales. SF-12 consisted of two components: a physical component summary (PCS) score and a mental component summary (MCS) score. These data for clinical characteristics were collected and evaluated by two independent rheumatologists (M.S.L. and L.H.C.) who had not participated in radiographic evaluations from a face-to-face questionnaire and medical records. Patients were evaluated clinically, using the Harris hip score (HHS) system by two orthopedic surgeons (Z.L. and Z.Y.X.) at the time of outpatient consultation. The HHS is based on the assessment of pain, function, deformity, and range of motion. On the 100-point scale, a score of 90 points or more is defined as an excellent outcome; 80–89 points, a good outcome; 70–79 points, a fair outcome; and 70 points or less, a poor outcome.

Laboratory data at enrollment, including human leukocyte antigen B27 (HLA-B27) status, level of serum erythrocyte sedimentation rate (ESR), C reactive protein (CRP) and highly sensitive CRP, were also measured.

### Radiographic measurement

**Fig. 1:**
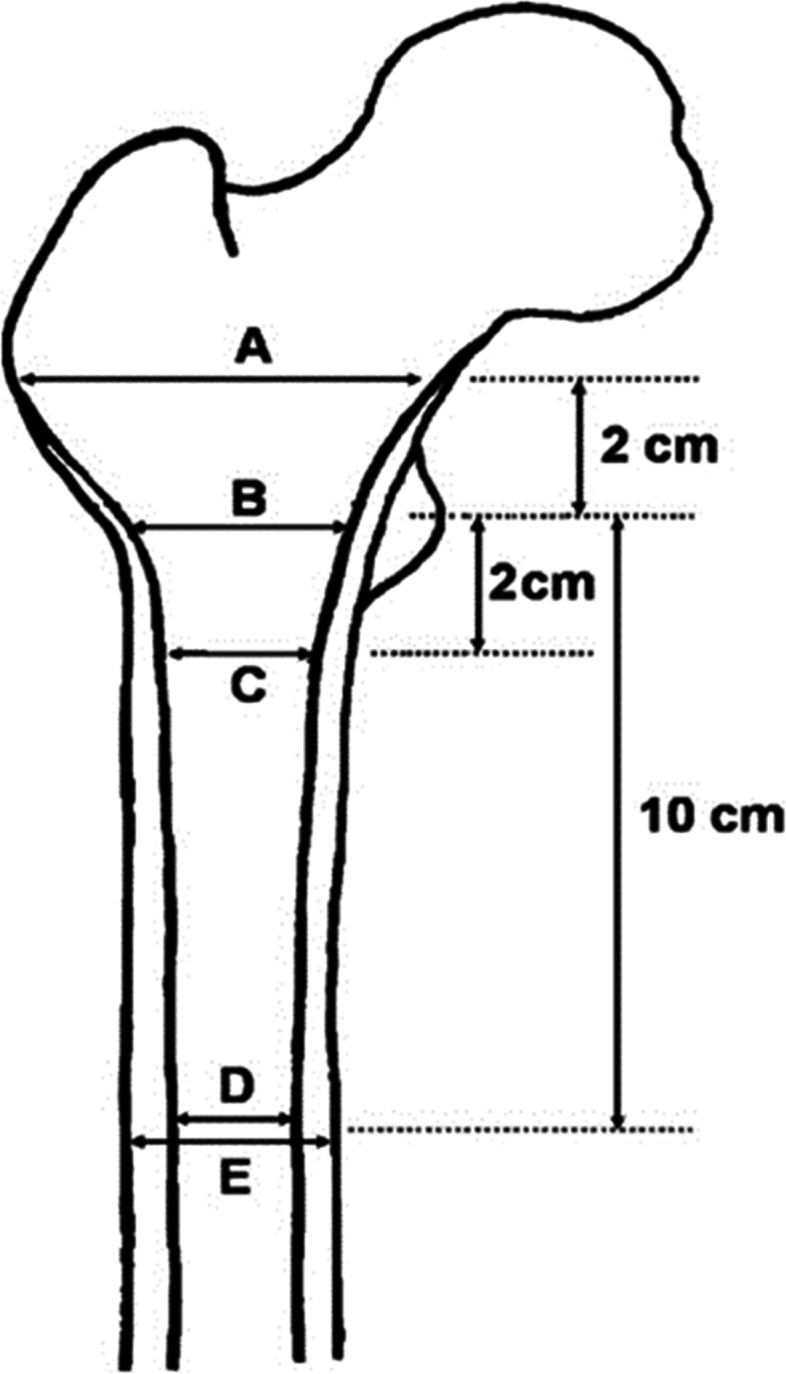
Morphological parameters of the proximal femur using anteroposterior radiographs of the pelvis: CFI = A/D; MCFI = A/C; CCR = D/B; CBR = D/E

For all patients, conventional digital anteroposterior (AP) radiographs of the pelvis were obtained from the electronic picture archiving and communication system (PACS) in our institute. The morphological parameters of the proximal femur included canal flare index (CFI) [29], metaphyseal canal flare index [MCFI] [30], canal to calcar ratio (CCR) [31] and canal bone ratio (CBR) [32] (Fig. 1). CFI was defined as a quotient of medullary canal width two centimeters above the center of lesser trochanter and medullary canal width at the isthmus of femur. MCFI was defined as a quotient of medullary canal width two centimeters above the center of lesser trochanter and medullary canal width two centimeters below the center of lesser trochanter. CCR was defined as a quotient of medullary canal width at the isthmus of femur and medullary canal width at the center of lesser trochanter. CBR was defined as a quotient of medullary canal width and outer bone diameter at the isthmus of femur.

According to the morphological classification system proposed by Noble et al. [29], the medullary canal of the proximal femur was classified into three types: a stovepipe type with a CFI < 3.0, a normal type with a CFI ≥ 3.0 and ≤ 4.7, and a champagne-fluted type with a CFI > 4.7.

These radiographs were evaluated by the same reviewer (B.T.) who was blinded from patients’ clinical information. Each parameter was measured twice with use of Mimics software (version 16.0; Nemaris, New York, USA) and averaged.

The severity of radiological hip involvement was assessed by using the bath ankylosing spondylitis radiology hip index (BASRI-Hip) system [33] on a five-point scale from 0 to 4 (0 = normal, no change; 1 = suspicious, possible focal joint space narrowing; 2 = minimal, circumferential joint space narrowing ≤2 mm; 3 = moderate, circumferential joint space narrowing> 2 mm, or bone-on-bone apposition of ≤2 cm; 4 = severe, bone deformity or bone-on-bone apposition of < 2 cm or THA). Consequently, all patients were categorized into two groups depending on the severity of hip involvement: absent, minimal or mild radiographic involvement (BASRI-Hip score = 0 or 1 or 2) (Group A) and moderate to severe radiographic involvement (BASRI-Hip score = 3 or 4) (Group B).

### Bone mineral measurement

DXA measurements were obtained using a Prodigy DXA scanner (GE, Lunar, Madison, WI, USA) and were analyzed using the manufacturer’s software. The involved hip was scanned in the supine position using posteroanterior projections. The T-score at the site of femoral neck, greater trochanter, Ward’s triangle plus the total hip measurement, was calculated on the basis of the Chinese reference database [34]. The femoral neck was selected as the region of interest in OP and osteopenia diagnosis. We used the diagnostic criteria established by the World Health Organization (WHO) in 1994. A T-score ≤ − 2.5 standard deviation (SD) indicates OP; − 2.5 SD < T-score < − 1.0 SD indicates osteopenia; T-score ≥ − 1.0 SD indicates normal.

QCT images were obtained by using Aquilion 64-slice CT scanner (Toshiba, Tokyo, Japan), and transferred to the Mindways QCT Pro work station for analysis (Mindways Software Inc., Austin, TX, USA). The scan parameters were as follows: 120 kV, 125 mA, scan time 0.5 s, table height 90 cm, pitch 0.938, field of view 40 cm, matrix 512*512, slice thickness 1 mm. All patients were placed supine, and scanned from iliac crest to 8 cm below the lesser trochanter. Solid Mindways QCT phantom was placed beneath the hip joint of patients when performing the scan. Images were analyzed using the CTXA HIP Version 4.2.3 Module of the Mindways QCT Pro software. The vBMD at the site of femoral neck, greater trochanter, intertrochanteric region plus the total hip measurement, was documented. The CTXA equivalent T-scores calculated from 2D projections of QCT data of the femoral neck were used for diagnosis of OP and osteopenia in accordance with the WHO criteria [34, 35]. Accordingly, the patients were divided into three groups according to the CTXA equivalent T-scores: OP group (T-score ≤ − 2.5 SD), osteopenia group (− 2.5 SD < T-score < − 1.0 SD) and normal group (T-score ≥ − 1.0 SD).

The laboratory examinations, radiographs, DXA scans were acquired on the same day in outpatient clinics and the QCT scans were conducted on the next day.

### Statistical analysis

All statistical analyses were performed using SPSS version 17.0 (IBM Inc., Chicago, IL). Descriptive analyses for categorical variables were based on percentage and for continuous variables on mean and SD. Demographic features, clinical characteristics, and radiographic parameters were compared using the Student t-test and Chi-square test between group A and B. These data were also compared using one-way ANOVA (including post hoc analysis) and nonparametric Kruskal-Wallis test among OP group, osteopenia group and normal group, respectively. The correlations between continuous variables and ordinal variables were determined using Pearson correlation analysis and Spearman rank correlation analysis by correlation coefficient (r), respectively. Statistical significance was defined as *p* < 0.05.

## Results

### Baseline demographics and clinical parameters

The patient demographics and clinical parameters were shown in Table 1. The results of BASRI-Hip score were as follows: 0 score in 13 hips (20%), 1 in 17 hips (26.2%), 2 in 9 hips (13.8%), 3 in 18 hips (27.7%) and 4 in 8 hips (12.3%). Consequently, group A consisted of 39 hips (0–2 score) and group B consisted of 26 hips (3–4 score).

**Table 1:** Patient demographics and clinical characteristics of AS patients on different stages of hip involvement

Characteristics	Total AS patients (*n* = 65)	Group A (*n* = 39)	Group B (*n* = 26)	*P* value
BMI	25.8 ± 4.7 (16.6–39.5)	25.9 ± 4.5 (17.7–38.9)	25.6 ± 5.0 (16.6–39.5)	0.851
Male gender, n (%)	56 (86.2%)	34 (87.2%)	22 (84.6%)	1.000
Age at onset (years)	23.3 ± 6.0 (14–39)	25.0 ± 5.9 (15–39)	20.9 ± 5.5 (14–34)	0.006
Age at outpatient visit (years)	33.3 ± 9.1 (17–54)	32.7 ± 7.5 (18–49)	34.2 ± 11.1 (17–54)	0.995
Duration of AS (years)	10.2 ± 9.4 (0–37)	8.0 ± 6.6 (0–24)	13.6 ± 11.8 (0–37)	0.131
Diagnosis delay (years)	5.6 ± 7.3 (0–33)	4.6 ± 6.2 (0–33)	7.1 ± 8.5 (0–27)	0.556
EAMs, n (%)				
Uveitis	4 (6.2%)	2 (5.1%)	2 (7.7%)	1000
IBD	4 (6.2%)	2 (5.1%)	2 (7.7%)	1.000
Family history, n (%)	14 (21.5%)	8 (20.5%)	6 (23.1%)	0.805
HLA-B27 positivity, n (%)	61 (93.8%)	37 (94.9%)	24 (92.3%)	1.000
Current use of NSAIDs, n (%)	43 (66.2%)	28 (71.8%)	15 (57.7%)	0.239
Current use of DMARDs, n (%)	35 (53.8%)	24 (61.5%)	11 (42.3%)	0.128
ESR (mm)	31.8 ± 20.3 (2–93)	27.9 ± 20.1 (2–91)	37.7 ± 19.6 (10–93)	0.020
CRP (mg/L)	26.8 ± 27.1 (1.9–133.0)	22.8 ± 26.6 (1.9–133.0)	32.7 ± 27.1 (2.2–107.0)	0.035
hsCRP (mg/L)	25.5 ± 28.7 (0.1–137.4)	22.3 ± 27.7 (0.1–131.5)	30.4 ± 30.0 (0.8–137.4)	0.111
BASDAI	3.8 ± 2.1 (0.4–8.4)	3.0 ± 1.9 (0.4–7.8)	4.8 ± 1.9 (0.4–8.4)	0.001
BASFI	2.1 ± 1.6 (0.2–6.3)	1.5 ± 1.0 (0.2–4.4)	3.2 ± 1.7 (0.5–6.3)	< 0.001
SF-12 PCS	37.5 ± 12.0 (16.5–58.4)	40.5 ± 11.8 (19.6–58.4)	33.1 ± 11.1 (16.5–55.1)	0.014
SF-12 MCS	39.3 ± 11.6 (17.1–57.8)	42.4 ± 11.3 (22.4–57.8)	34.9 ± 10.8 (17.1–54.3)	0.009
ASQOL	6.4 ± 4.5 (0–17)	5.0 ± 4.1 (0–14)	8.4 ± 4.3 (2–17)	0.002
HHS	75.7 ± 19.8 (24–100)	87.5 ± 9.6 (65–100)	58.1 ± 18.1 (24–95)	< 0.001
BASRI-Hip	1.9 ± 1.4 (0–4)	0.9 ± 0.8 (0–2)	3.3 ± 0.5 (3–4)	< 0.001

Group A represents the hips with absent, minimal or mild radiographic involvement (BASRI-Hip score = 0 or 1 or 2) and Group B represents the hips with moderate to severe radiographic involvement (BASRI-Hip score = 3 or 4)

The value of continuous variables was presented as mean ± standard deviation and the categorical variables were based on presented as number plus percentage

*Abbreviations*: *AS* Ankylosing spondylitis, *BMI* Body mass index, *EAMs* Extra-articular manifestations, *IBD* Inflammatory bowel disease, *NSAIDs* Nonsteroidal anti-inflammatory drugs, *DMARDs* Disease modifying anti-rheumatic drugs, *ESR* Erythrocyte sedimentation rate, *CRP* C reactive protein, *hsCRP* High sensitive C reactive protein, *BASDAI* Bath ankylosing spondylitis disease activity index, *BASFI* Bath ankylosing spondylitis functional index, *SF-12 PCS* short form-12 physical component summary, *SF-12 MCS* Short form-12 mental component summary, *ASQOL* Ankylosing spondylitis quality of life, *HHS* Harris hip score, *BASRI-Hip* The bath ankylosing spondylitis radiology hip index

### Diagnosis consistence between DEXA and QCT

The average BMD of femoral neck measured by DXA was 0.87 ± 0.18 g/cm^2^ (range, 0.47–1.21 g/cm^2^) at the site of femoral neck, 0.69 ± 0.14 g/cm^2^ (range, 0.38–0.98 g/cm^2^) at greater trochanter, 0.74 ± 0.15 g/cm^2^ (range, 0.42–1.10 g/cm^2^) at Ward’s triangle. The average trabecular BMD measured by QCT was 136.38 ± 25.58 mg/cm^3^ (range, 82.10–200.60 mg/cm^3^) at the site of femoral neck, 121.02 ± 21.19 mg/cm^3^ (range, 78.30–170.40 mg/cm^3^) at greater trochanter,129.23 ± 27.55 mg/cm^3^ (range, 70.40–187.30 mg/cm^3^) at intertrochanteric region. Correspondingly, the CTXA equivalent T-score from QCT was − 1.11 ± 1.12 (range, − 3.38-2.59) at the site of femoral neck, − 1.51 ± 1.06 (range, − 3.71-0.44) at greater trochanter, − 1.35 ± 1.41 (range, − 4.46-1.59) at intertrochanteric region.

The detection rate for osteopenia was 29.2% for DXA and 43.1% for QCT at the site of femoral neck, respectively (*p* = 0.100). The detection rate for OP was 12.3% for DXA and 12.3% for QCT at total hip, respectively (*p* = 1.000).

### Intergroup comparisons

The differences in demographics, clinical parameters between group A and group B are presented in Table 1. Compared with patients in group A, the patients in group B had a significantly lower age at disease onset (20.9 ± 5.5 vs 25.0 ± 5.9, *p* = 0.006), a higher level of ESR (37.7 ± 19.6 vs 27.9 ± 20.1, *p* = 0.020), a higher level of CRP (32.7 ± 27.1 vs 22.8 ± 26.6, *p* = 0.035), a higher BASDAI (4.8 ± 1.9 vs 3.0 ± 1.9, *p* = 0.001), a higher BASFI (3.2 ± 1.7 vs 1.5 ± 1.0, *p* < 0.001), a lower SF-12 PCS (33.1 ± 11.1 vs 40.5 ± 11.8, *p* = 0.014), a lower SF-12 MCS (34.9 ± 10.8 vs 42.4 ± 11.3, *p* = 0.009), a lower HHS (58.1 ± 18.1 vs 87.5 ± 9.6, p < 0.001).

The distribution of CFI was as follows: 17 hips (26.2%) with a stovepipe type (CFI < 3.0), 43 hips (66.2%) with a normal type (3.0 ≤ CFI ≤ 4.7), and 5 hips (7.6%) with a champagne-fluted type (CFI > 4.7). We also compared the morphological parameters of the proximal femur, including CFI, MCFI, CCR, CBR between group A and B, and found no significant differences (Table 2).

**Table 2:** Radiographic parameters of AS patients on different stages of hip involvement

Parameters	Total AS patients (*n* = 65)	Group A (*n* = 39)	Group B (*n* = 26)	*P* value
CFI	3.52 ± 0.79 (1.70–5.77)	3.53 ± 0.70 (2.12–4.96)	3.50 ± 0.92 (1.70–5.77)	0.698
MCFI	2.19 ± 0.29 (1.33–3.04)	2.20 ± 0.31 (1.33–3.04)	2.19 ± 0.26 (1.76–2.66)	0.779
CCR	0.48 ± 0.11 (0.30–0.86)	0.48 ± 0.10 (0.32–0.74)	0.48 ± 0.12 (0.30–0.86)	0.883
CBR	0.46 ± 0.08 (0.31–0.65)	0.45 ± 0.07 (0.31–0.58)	0.48 ± 0.09 (0.32–0.65)	0.194

Group A represents the hips with absent, minimal or mild radiographic involvement (BASRI-Hip score = 0 or 1 or 2) and Group B represents the hips with moderate to severe radiographic involvement (BASRI-Hip score = 3 or 4)

*Abbreviations*: *AS* Ankylosing spondylitis, *CFI* Canal flare index, *MCFI* Metaphyseal canal flare index, *CCR* Canal to calcar ratio, *CBR* Canal bone ratio

We compared the trabecular vBMD (CTXA equivalent T-score) between group A and B and found significant differences at each of three regions: − 0.82 ± 1.05 vs − 1.55 ± 1.23, *p* = 0.013 (femoral neck); − 0.80 ± 1.29 vs − 2.19 ± 1.21, *p* < 0.001 (intertrochanteric region); − 1.12 ± 0.84 vs − 2.09 ± 1.14, *p* = 0.001 (greater trochanter). We found a statistically significant correlation between the vBMD (CTXA equivalent T-score) and the morphological and clinical parameters as shown in Table 3.

**Table 3:** Correlations between trabecular CTXA equivalent T-scores and clinical and radiographic parameters of AS patients on different stages of hip involvement

	Male gender	Age at outpatient visit	Disease duration	BASRI-hip	HHS	CBR
Trabecular CTXA equivalent T-scores (femoral neck)	− 0.286 (0.021)	− 0.27 (0.029)	−0.311 (0.012)	− 0.261 (0.035)	0.244 (0.050)	− 0.319 (0.010)
Trabecular CTXA equivalent T-scores (greater trochanter)	−0.197 (0.116)	− 0.096 (0.446)	−0.187 (0.135)	− 0.314 (0.011)	0.265 (0.033)	− 0.418 (0.001)
Trabecular CTXA equivalent T-scores (intertrochanteric region)	−0.283 (0.022)	− 0.061 (0.632)	−0.191 (0.128)	− 0.326 (0.008)	0.261 (0.036)	− 0.446 (< 0.001)

Results are presented as coefficient of correlation and *P* value in brackets

*Abbreviations*: *vBMD* Volumetric bone mass density, *BASRI-Hip* The bath ankylosing spondylitis radiology hip index, *HHS* Harris hip score, *CBR* Canal bone ratio

The clinical and radiographic parameters among OP group, osteopenia group and normal group were also compared and the significant differences existed in the parameters as listed below (Table 4). The BMI was significantly lower in OP group than that in normal group (22.4 ± 4.3 vs 27.0 ± 4.3, *p* = 0.013); the age at disease onset was significantly lower in OP group than that in normal group (18.5 ± 3.8 vs 25.0 ± 6.1, *p* = 0.006); The HHS was significantly lower in osteopenia group than that in normal group (72.5 ± 27.9 vs 83.6 ± 13.1, *p* = 0.004).

**Table 4:** Intergroup comparisons of clinical and radiographic parameters between OP group, osteopenia group and normal group

Characteristics	OP group (*n* = 8)	Osteopenia group (*n* = 28)	Normal group (*n* = 29)	*P* value
BMI	22.4 ± 4.3 (17.3–28.5)	25.5 ± 4.8 (16.6–39.5)	27.0 ± 4.3 (17.5–38.9)	0.040
Male gender, n (%)	8 (100%)	25 (89.3%)	23 (79.3%)	0.271
Age at outpatient visit (years)	28.3 ± 12.5 (17–54)	35.3 ± 8.8 (21–54)	32.9 ± 7.9 (21–54)	0.143
Age at onset (years)	18.5 ± 3.8 (14–24)	23.0 ± 5.9 (15–38)	25.0 ± 6.1 (15–39)	0.021
Duration of AS (years)	10.0 ± 12.7 (0–37.0)	12.5 ± 10.5 (0.5–37.0)	8.1 ± 6.8 (0–22.0)	0.214
Diagnosis delay (years)	4.6 ± 6.8 (0.0–18.0)	6.11 ± 7.23 (0–27.0)	5.3 ± 7.4 (0–33.0)	0.855
EAMs, n (%)				
Uveitis	0	2 (7.1%)	2 (6.9%)	0.744
IBD	0	2 (7.1%)	2 (6.9%)	0.744
Family history, n (%)	3 (37.5%)	7 (25.0%)	4 (13.8%)	0.302
HLA-B27 positivity, n (%)	8 (100%)	26 (92.9%)	27 (93.1%)	0.744
Current use of NSAIDs, n (%)	5 (62.5%)	16 (57.1%)	22 (75.9%)	0.325
Current use of DMARDs, n (%)	5 (62.5%)	12 (42.9%)	18 (62.1%)	0.308
ESR (mm)	30.9 ± 21.2 (2–65)	35.8 ± 21.6 (3–93)	28.3 ± 18 (8–91)	0.383
CRP (mg/L)	19.2 ± 10.9 (3.5–35.2)	33.4 ± 32.9 (3.2–133.0)	22.5 ± 22.8 (1.9–100.0)	0.222
hsCRP (mg/L)	16.8 ± 13.2 (0.1–34.2)	31.9 ± 34.9 (0.8–137.4)	21.8 ± 24.2 (0.2–114.6)	0.277
BASDAI	3.9 ± 1.8 (1.4–5.8)	4.3 ± 2.0 (0.6–8.4)	3.1 ± 2.1 (0.4–7.8)	0.094
BASFI	2.4 ± 1.7 (0.5–5.5)	2.4 ± 1.7 (0.3–6.3)	1.8 ± 1.3 (0.2–4.0)	0.269
SF-12 PCS	35.1 ± 15.5 (16.5–58.4)	34.9 ± 11.6 (18.2–55.4)	40.7 ± 11.0 (18.5–55.9)	0.158
SF-12 MCS	36.1 ± 15.9 (17.1–55.4)	37.4 ± 11.6 (20.3–57.9)	42.2 ± 10.0 (21.4–55.9)	0.197
ASQOL	7.5 ± 5.9 (2–17)	7.0 ± 4.2 (1–16)	5.5 ± 4.2 (0–14)	0.344
HHS	72.5 ± 27.9 (24–100)	68.6 ± 20.8 (36–100)	83.6 ± 13.1 (48–99)	0.013
BASRI-Hip	1.9 ± 1.3 (0–3)	2.5–1.3 (0–4)	1.2 ± 1.2 (0–4)	0.002
CFI	3.4 ± 1.0 (1.7–4.8)	3.5 ± 0.9 (2.2–5.8)	3.6 ± 0.7 (2.1–5.0)	0.763
MCFI	2.1 ± 0.3 (1.8–2.7)	2.2 ± 0.2 (1.8–2.6)	2.2 ± 0.3 (1.3–3.0)	0.766
CBR	0.4 ± 0.1 (0.3–0.6)	0.5 ± 0.1 (0.3–0.7)	0.4 ± 0.1 (0.3–0.6)	0.094
CCR	0.5 ± 0.2 (0.36–0.9)	0.5 ± 0.1 (0.3–0.8)	0.5 ± 0.1 (0.3–0.7)	0.768

The value of continuous variables was presented as mean ± standard deviation and the categorical variables were based on presented as number plus percentage

*Abbreviations*: *OP* Osteoporosis, *BMI* Body mass index, *AS* Ankylosing spondylitis, *EAMs* Extra-articular manifestations, *IBD* Inflammatory bowel disease, *NSAIDs* Nonsteroidal anti-inflammatory drugs, *DMARDs* Disease modifying antirheumatic drugs, *ESR* Erythrocyte sedimentation rate, *CRP* C reactive protein, *hsCRP* High sensitive C reactive protein, *BASDAI* Bath ankylosing spondylitis disease activity index, *BASFI* Bath ankylosing spondylitis functional index, *SF-12 PCS* Short form-12 physical component summary, *SF-12 MCS* Short form-12 mental component summary, *ASQOL* Ankylosing spondylitis quality of life, *HHS* Harris hip score, *BASRI-Hip* The bath ankylosing spondylitis radiology hip index

## Discussion

In the current study, DXA and QCT techniques were used as the evaluation tools for BMD measurement in patients with AS on different stages of hip involvement. The femoral neck was selected as the site of region of interest (ROI). In a longitudinal follow-up study by DXA method, Deminger et al. found that BMD decreased at the femoral neck and increased at the lumbar spine in AS patients after 5 years, both in the AP and the lateral projections. They suggested that the best site to assess bone loss in AS patients is the femoral neck [23]. Literatures published previously have discussed on the comparisons between DXA and QCT applications in specific patient population, including elderly men [36] and postmenopausal women [26]. The main advantage of QCT in the evaluation of OP and osteopenia for AS patients relies on its inherent ability of eliminating the interference of syndesmophyte formation [17, 25, 26, 37]. In the current study, QCT had the same detection rate for OP (12.3% vs 12.3%). Interestingly, compared with DXA, QCT had the higher detection rate for osteopenia (44.6% vs 29.2%) for QCT, although the differences had no statistical significance (*p* = 0.100). The results suggested that the syndesmophyte formation around the proximal femur possibly affect the accuracy of DXA based on two-dimensional projection.

In our series, the percentage of stovepipe type (CFI < 3.0) was only 26.2%. There were also no significant differences between group A and B as well as among OP, osteopenia, and normal group regarding these morphological parameters of the proximal femur, including CFI, MCFI, CCR, CBR. Our results were inconsistent with previous studies. Some authors reported a higher percentage of Dorr type C femurs in AS patients with advanced stage of hip involvement and cemented femoral stems were recommended in AS patients with severe OP to ensure the reliable fill of the prosthesis to the canal [38, 39]. In a Chinese cadaver research, Yeung et al. found the value of CBR showed a strong correlation with the DXA T score (*r* = − 0.71, *P* < 0.001) and the best overall performance in diagnosing OP with receiver operating characteristic (ROC) curve analysis [40]. The proximal femur was likely to be osteoporotic if the canal bone ratio was 0.49 or higher. We also found a similar negative correlation between CBR and vBMD at each of three regions. A further investigation with a larger sample size is anticipated.

A series of studies have shown the negative relationship between BMD and disease activity parameters (ESR, CRP and BASDAI), indicating that bone loss in AS is predominantly the consequence of inflammatory process [13, 14, 18, 37]. Grazio et al. [14] investigated 80 established AS patients and found that there was a significant negative correlation of bone density T scores with CRP and ESR, which was reflected more obviously at proximal femur than at lumbar spine. There were also significant differences in ESR, BASDAI, BASFI and global health among normal, osteopenia and OP group. Their results indicated an association of low BMD with high disease activity in patients with AS. Femoral BMD seemed to be more associated with disease activity and functional ability than lumbar spine BMD. Unfortunately, as far as we know, no previous studies have discussed the relationship between hip BMD and clinical and radiographic status of involved hip on different stages in AS patients.

In the current study, inconsistent with previous results, we did not find any correlations between hip vBMD and inflammatory markers including ESR, CRP and hsCRP as well as disease activity score (BASDAI). Our explanations Our explanations are as follows: first, the sample size was relatively small (*n* = 65). Second, the hips in our study were on different stages of hip involvement. Once the hip involvement has progressed to an end stage, the inflammatory marker and disease activity tend to be normal. In contrast, the vBMD in intertrochanteric region and greater trochanter was negatively correlated with BASRI-hip score (*r* = − 0.326, *r* = − 0.314, respectively), which represented the degree of radiographic involvement. The vBMD at femoral neck was negatively correlated with disease duration(*r* = − 0.311). In the intergroup comparisons, the hips with moderate to severe radiographic involvement had a significantly lower trabecular vBMD at femoral neck, intertrochanteric region and greater trochanter, compared with those with absent, minimal or mild involvement. Correspondingly, osteopenia group had a significantly lower HHS than that in normal group (72.5 ± 27.9 vs 83.6 ± 13.1, *p* = 0.004). These results indicated that the low trabecular bone density was associated with the duration of disease progression and did have a negative impact on the functional status of involved hip although a large proportion of hips were still on the stage of osteopenia (*n* = 28).

There are several limitations to note in the present study. First, it was a single-center study with a small sample size (*n* = 65). Second, as a cross-sectional research design, we were unable to adequately evaluate the dynamic change of hip BMD and its impact on the functional status of involved hip. We also failed to dynamically trace the change of disease activity and inflammatory markers including ESR and CRP. Third, the classification system used for describing the degree of hip involvement in these AS patients were BASRI-Hip score based on conventional radiographs. Obviously, this system lacks enough sensitivity and accuracy for evaluating the involvement of hip, especially in the early stage. The magnetic resonance imaging system can be introduced as a reliable tool for detecting the early involvement of hip in AS patients.

## Conclusions

In conclusion, our results suggested that OP and osteopenia were common among patients with AS. The low trabecular bone density at the site of hip was associated with the duration of disease progression and the degree of hip involvement. Meanwhile, it also had a relationship with the functional status of involved hip although our study failed to confirm a significant association between hip vBMD and inflammatory markers as well as disease activity.

## Data Availability

The datasets used and/or analyzed during the current study are available from the corresponding author on reasonable request.

## Abbreviations

AP: Anteroposterior
AS: Ankylosing spondylitis
ASQoL: Ankylosing Spondylitis Quality of Life
BASDAI: Bath ankylosing spondylitis disease activity index
BASFI: Bath ankylosing spondylitis functional index
BASRI-Hip: bath ankylosing spondylitis radiology hip index
BMD: Bone mineral density
BMI: Body mass index
CBR: Canal bone ratio
CCR: Canal to calcar ratio
CFI: Canal flare index
CRP: C reactive protein
DMARDs: Disease modifying antirheumatic drugs
DXA: Dual-energy x-ray absorptiometry
EAMs: Extra-articular manifestations
ESR: Erythrocyte sedimentation rate
HHS: Harris hip score
HLA-B27: Human leukocyte antigen B27
hsCRP: High sensitive C reactive protein
IBD: Inflammatory bowel disease
MCFI: Metaphyseal canal flare index
NSAIDs: Nonsteroidal anti-inflammatory drugs
OP: Osteoporosis
PACS: Picture archiving and communication system
PROs: Patient-reported outcomes
QCT: Quantitative computed tomography
ROC: Receiver operating characteristic
ROI: Region of interest
ROM: Range of motion
SD: Standard deviation
SF-12 MCS: Short form-12 mental component summary
SF-12 PCS: Short form-12 physical component summary
THA: Total hip arthroplasty
vBMD: Volumetric bone mineral density
WHO: World Health Organization

## Declarations

All methods were carried out in accordance with relevant guidelines and regulations.
